# Race/ethnic disparities in early childhood BMI, obesity and overweight in the United Kingdom and United States

**DOI:** 10.1038/ijo.2014.171

**Published:** 2014-10-14

**Authors:** A Zilanawala, P Davis-Kean, J Nazroo, A Sacker, S Simonton, Y Kelly

**Affiliations:** 1 Department of Epidemiology and Public Health, University College London, London, UK; 2 Institute for Social Research, University of Michigan, Ann Arbor, MI, USA; 3 School of Social Sciences, The Cathie Marsh Centre for Census and Survey Research, University of Manchester, Manchester, UK

## Abstract

**Objective:**

Racial/ethnic patterning in the risk of obesity and overweight has been observed in early childhood; however, little research has compared these disparities between the United Kingdom (UK) and United States (US) using detailed ethnic classifications. We use comparable nationally representative cohort studies to examine racial/ethnic disparities in mean body mass index (BMI) and in the odds of obesity/overweight in the UK and US. The contribution of sociodemographic, cultural and family routine factors are assessed.

**Methods:**

Data on BMI, obesity and overweight in 5-year-old children from the MCS (Millennium Cohort Study) and ECLS-B (Early Childhood Longitudinal Study, Birth Cohort) were examined. We investigated race/ethnic disparities in mean BMI and in the odds of obesity and overweight, as compared to normal weight. We assessed the independent contribution of sociodemographic, cultural and family routine factors to observed disparities.

**Results:**

In the UK, after adjustment for sociodemographic, cultural and family routine factors and maternal BMI, we found Black Caribbean children to have higher odds ratio (OR=1.7, confidence interval (CI)=1.1–2.6), Pakistani children to have lower odds of obesity (OR=0.60, CI=0.37–0.96) and Black African children were more likely to be overweight (OR=1.40, CI=1.04–1.88). In the US, in fully adjusted models, there were no race/ethnic disparities in children's odds of obesity and overweight.

**Conclusion:**

Disparities for Bangladeshi children in the UK and Mexican, other Hispanic and American Indian children in the US can be explained by socioeconomic disadvantage, whereas a range of cultural and family characteristics partially explain disparities for other groups in the UK. Future public health initiatives focused on reducing risk of overweight and obesity should consider the diverse socioeconomic and cultural profiles of all race/ethnic groups.

## Introduction

Over the last several decades, the prevalence of child obesity has increased.^1^ High body mass index (BMI) in early childhood is associated with onset of chronic conditions, including diabetes and hypertension^2^ during childhood, and children who are overweight are more likely to be obese as adults and to have obesity-related illnesses.^3^ There are stark race/ethnic disparities in child adiposity. In the United Kingdom (UK), Black African, Black Caribbean and South Asian children are more likely to be overweight or obese than White children.^4, 5, 6, 7^ In the United States (US), higher rates of obesity are documented among Black, Hispanic and American Indian children relative to White children.^8, 9, 10, 11, 12, 13^ Previous research emphasizes the importance of understanding racial and ethnic disparities at early ages and use of detailed racial and ethnic classifications.^6,10^

Prior studies have linked socioeconomic, cultural, nutritional and family routine factors to race/ethnic disparities in high BMI.^10,14, 15, 16^ Income and education are thought to be protective factors as higher levels of both are linked to knowledge of healthy food choices and children's physical activity,^17^ and are associated with lower risk of childhood overweight or obesity in the UK and US.^1,5,10^ However, some studies suggest that racial/ethnic minority children from households with low income and whose parents have low educational attainment may be less likely to be overweight or obese than their socioeconomically advantaged racial/ethnic minority counterparts.^1,4,18^ Evidence for the influence of maternal employment on children's BMI is mixed.^19,20^ Markers of cultural location, such as the language spoken at home and age of parental migration, are linked to healthy food choices and attitudes, but such factors may be disadvantageous if they block important information on nutrition and exercise. Cultural factors, such as migration status and English proficiency, have been linked to higher BMI and risk of obesity among children in the US,^21,22^ yet evidence from the UK suggests no association between age at migration and children's risk of overweight.^4^ Racial and ethnic differences in family routines, such as regular bedtimes and child nutrition, have been observed and these factors are associated with risk of obesity in children.^4,19,23^ Concurrent maternal BMI is also posited to be linked to child obesity and may indicate suboptimal dietary choices at home. Maternal obesity by race/ethnicity and mother's BMI is in turn a predictor of child overweight and obesity.^5,24^

Studies have highlighted the importance of examining the contribution of child and family characteristics in explaining the risk of overweight and obesity in early childhood.^5,16^ In addition, research examining race/ethnic disparities in children's health markers has supported using a detailed ethnic classification.^25, 26, 27^ Comparative analyses are important, since, despite the cultural similarities between the UK and US, there is considerable variation in social systems, migration patterns and historical and contemporaneous race/ethnic relations.^28, 29, 30^ Recent comparative work has found race/ethnic disparities in child overweight in the US and England,^4,31^ but did not consider a detailed ethnic classification in the UK and US. Aggregating race and ethnic groups in both countries obscures their heterogeneity in socioeconomic, migratory and health profiles.^32^ Furthermore, the studies have not included language spoken at home and markers of child nutrition and family routines that may help to explain observed disparities.

Our study addresses these gaps by using a detailed ethnic classification in both the UK and US analyses and by accounting for a rich set of socioeconomic, cultural and family and child characteristics in an attempt to explain observed disparities. We use data from two comparable nationally representative cohort studies to examine racial/ethnic patterning in mean BMI, overweight and obesity and the factors underlying any differences in this patterning in the UK and US.

**Figure 1:**
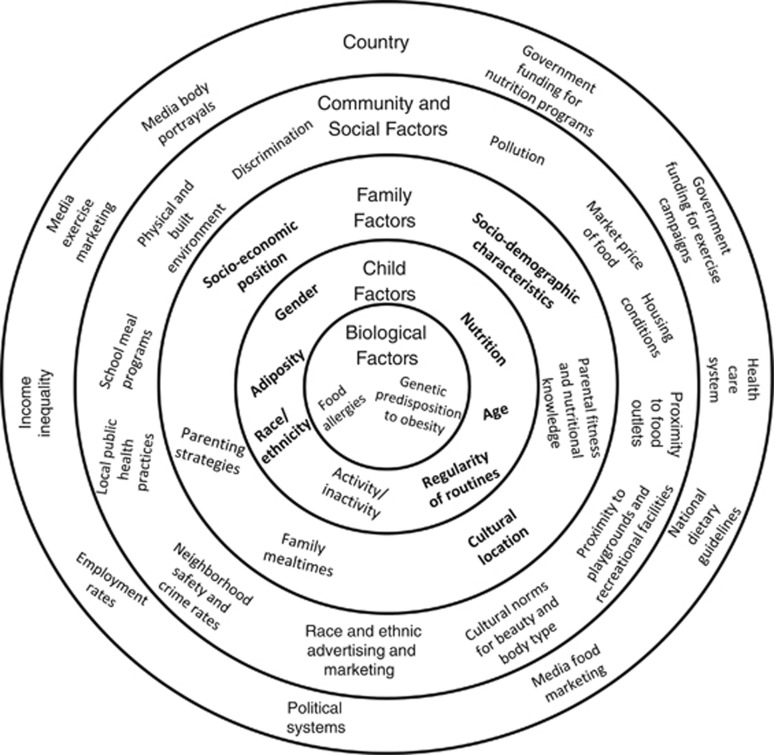
Measured and unmeasured factors influencing race/ethnic disparities in child adiposity. Notes: Factors in bold are included in analyses. Nutrition assessed by fruit consumption. Regularity of routines indicated by sleep schedules. Socioeconomic position measured by parental education and household income. Sociodemographic factors include maternal employment status and single parenthood. Indicators of cultural location are maternal migration status and English as the home language. Parental fitness and nutritional knowledge indicated by maternal body mass index (BMI). Child adiposity measured by BMI.

We hypothesize (1) children of minority status in the UK and US to be at greater risk of overweight and obesity in early childhood; (2) socioeconomic disadvantage to be a risk factor for childhood overweight and obesity; (3) a less traditional cultural location to be protective; and (4) irregular family routines to be associated with greater risk. In addition, maternal BMI will be highly predictive of child overweight and obesity. Figure 1 summarizes measured and unmeasured factors influencing the risk of childhood overweight and obesity (adapted from Harrison).^33^

## Materials and Methods

### Millennium Cohort Study

The Millennium Cohort Study (MCS) is a cohort study of 18 552 children born in the UK between 2000 and 2002. It is representative of infants who were alive and residing in the UK at 9 months of age and who were eligible for Child Benefit (a nearly universal monetary benefit).^34^ The sample was clustered at the electoral ward level, with an oversample of disadvantaged residential areas and areas with a high proportion of ethnic minority residents. The first interviews were at 9 months of age, and follow-up sweeps were conducted at ages 3, 5, 7 and 11. New families were recruited at the second sweep increasing the number of families who have ever participated in the MCS to 19 244. To enable comparison, with available US data we used measures collected during the third sweep of interviews which were conducted through home visits when the cohort child was ~5 years of age. During the interview, anthropometric measurements were taken and questions were asked about sociodemographic circumstances, cultural traditions and family routines. The main respondent was usually the mother (98%) and information about their partners was collected through separate interviews.

### Early Childhood Longitudinal Study, Birth Cohort

The Early Childhood Longitudinal Study, Birth Cohort (ECLS-B) is a nationally representative sample of children born in the US in 2001. The sample, drawn from birth certificates, is representative of 9-month infant survivors born to mothers who were at least 15 years of age and who did not place their child into adoption.^35^ A sample of 14 000 births yielded a study cohort of 10 700 children whose parents were successfully interviewed when the children were 9 months old. The study oversampled children who were American Indian, Chinese and other Asian. Parent interviews were at 9 months, 2 years, 4 years and upon entry into kindergarten. The analyses presented in this paper use data collected from children when they entered kindergarten, either in fall 2006 or 2007, aged ~5 years. Home interviews collected information on children's health, sociodemographic characteristics and home environment. The vast majority of these interviews (95%) were conducted with the child's mother.^36^

The MCS and ECLS-B are comparable: participants were born contemporaneously; the samples are nationally representative; data were collected around the same age at follow-up; and datasets include a range of similar explanatory factors.

### Anthropometric measurements

In both the MCS and ECLS-B, during home visits, interviewers measured children's height and weight using standardized protocols. BMI was categorized into three mutually exclusive categories (normal, overweight and obese) using age- and gender-specific thresholds as defined by the International Obesity Task Force.^37^ These thresholds are linked to adult cut points of 25 and 30 kg m^−2^ for adult overweight and obesity, respectively. Children who were underweight (nearly 2% in the MCS and ECLS-B) were included in the normal weight group for analyses. Separate analyses (not shown) excluding underweight children did not affect substantive results.

### Race/ethnicity

Racial and ethnic categories were constructed using mother's reports of her child's race/ethnicity and were based on census categories within each country. In the UK, the groups used for analysis were: White, Indian, Pakistani, Bangladeshi, Black Caribbean (including mixed White and Black Caribbean), Black African (including mixed White and Black African) and other. For the US, the groups were White, Black, Mexican, other Hispanic, Asian Indian, east Asian, southeast Asian, American Indian and other. For both the UK and US, ‘other' includes mixed racial/ethnic groups and racial/ethnic minority groups that could not be categorized into any of the otherwise defined groups.

### Explanatory factors

The potential contribution to racial and ethnic disparities in childhood overweight/obesity of sociodemographic characteristics; cultural tradition and family routine markers; an indicator of child nutrition; and maternal BMI were assessed. Sociodemographic characteristics were child's gender and age (centered at the mean), mother's employment status (working full-time, working part-time, not working or not present in the household), single parenthood and markers of socioeconomic position, which were equivalized household income in quintiles^38^ and highest parental educational qualification. Measures of education that are meaningful in each country but not directly comparable were used. In the UK, the education variable was categorized into five levels: less than O level (ordinary level), O level, A level (advanced level), degree or higher and overseas qualifications. In the US, the education variable consisted of four categories: less than high school, high school/general education diploma, some college and bachelor's degree or higher. Equivalized income and education categories are similar to those used in previous literature comparing the UK and US.^39, 40, 41^ Cultural tradition markers were a binary indicator of English as the primary language spoken at home and a categorical variable for maternal migration status (first, second and third generation or more). Family routine variables were a dichotomous measure of regular bedtime on weekdays (always/usually or sometimes/never) and a categorical variable for bedtimes (not regular, before 1930, 1930–1959, 2000–2029, 2030–2059 and 2100 h and later).^42^ Child nutrition is indicated by the number of portions of fruit eaten per day (none, one, two or three plus). Maternal BMI was also taken into account.

### Sample

The MCS analytic sample was 18 280 and the ECLS-B analytic sample was 8850 after multiply imputing missing values and observations due to item non-response and attrition. We excluded multiple births and observations for which height and weight were not assessed at any interview. The rate of missingness in the MCS was between 0 and 32% for explanatory factors, of which mother's BMI had the highest rate of missingness (Supplementary Appendix Table 1). The range in rate of missingness was higher in the ECLS-B (0–40%) with over half of the explanatory factors missing at the rate of 40%. In the MCS, mothers who were missing information on their children's BMI were less likely to be White, more likely to have low income and lower educational attainment and more likely to be unemployed than those who had complete information on children's BMI (Supplementary Appendix Table 2). Socioeconomic disadvantage was also evident among mothers in ECLS-B who were missing information on children's BMI as compared to those with complete information on BMI, but the magnitude of difference between mothers with incomplete and complete information was smaller as compared to differences in the MCS (Supplementary Appendix Table 3).

For both datasets the imputation model included all explanatory factors, auxiliary variables measuring sociodemographic characteristics from previous interviews and design variables to account for the clustered nature of the data. By using STATA^43^ and SAS, we used multiple imputation techniques, which account for uncertainty about missing values by imputing several values for each missing data point (with variability due to both sampling error and model uncertainty).^44^ We imputed 25 datasets and consolidated results from all imputations for analyses using Rubin's^45^ combination rules. Post-imputation diagnostics did not reveal large deviations between estimates from complete case and imputed analyses. The imputed estimates were slightly more efficient than complete case analyses, but the coefficients in the imputed analyses had the same direction and significance as ones in complete case analyses. We tested the sensitivity of these results by excluding cases with imputed values on the dependent variable and only selecting mothers who were interviewed. Results were robust in these two subsamples. Imputation literature recommends using imputation on all variables when the imputation model includes auxiliary variables, as it does in our analyses, because such variables provide extra information on the outcome variables.^46^ We present results from the imputed samples.

### Analytical approach

In order to understand the race/ethnic disparities in children's health at age 5, we investigated the independent contribution of sociodemographic, cultural and family routine factors in predicting children's mean BMI and odds of overweight and obesity across racial/ethnic groups. In the base model, we present estimates of racial/ethnic differences controlling only for child age and gender. Then we assessed the importance of explanatory factors by separately adjusting for five sets of covariates: model 1 adjusts for sociodemographic characteristics; model 2 adjusts for sociodemographic and cultural factors; model 3 adjusts for sociodemographic and family routine characteristics; model 4 adjusts for sociodemographic characteristics and a marker of child nutrition; model 5 adjusts for sociodemographic characteristics and maternal BMI; and model 6 simultaneously controls for all covariates. For models predicting mean BMI, we use ordinary least squares regressions; for models predicting normal weight, overweight and obesity, we use multinomial logistic regression models and present odds ratios. All analyses use the largest group (White children) as the reference group.

All analyses used sample weights from the 9-month interviews in both datasets to adjust for unequal probability of being sampled and the stratified and clustered sample design. Survey procedures in STATA and SAS produced estimates and s.e. adjusting for sample design. Reported sample sizes were rounded to the nearest 50 in ECLS-B analyses as per data user requirements.

## Results

Tables 1 and 2 show children's BMI, obesity and the distribution of explanatory factors according to children's racial/ethnic group for the UK and US, respectively. In the UK, Bangladeshi, Black Caribbean and Black African children were most likely to be obese. In the US, obesity rates were higher than those in the UK; Mexican, other Hispanic and American Indian children were most likely to be obese.

In both countries, socioeconomic disadvantage was associated with racial and ethnic minority groups. In particular, Pakistani, Bangladeshi, Black Caribbean and Black African children in the UK, and Black, Mexican and American Indian children in the US were most likely to live in households with annual incomes in the lowest quintile. In the UK, mothers of Pakistani and Bangladeshi children had the least educational attainment. In the US, mothers of Black, Mexican and American Indian children had the lowest college completion rates.

There was heterogeneity in the distribution of other explanatory factors by race/ethnicity. In the UK, mothers of Indian, Pakistani and Bangladeshi children were most likely to speak a language other than English or in addition to English at home. This was true for mothers of Mexican and other Hispanic children in the US. Bangladeshi children in the UK and East Asian children in the US were most likely to have bedtimes after 2100 h. Bangladeshi children in the UK, and Black and American Indian children in the US were least likely to eat any fruit per day. High maternal BMI was evident among mothers of Black African children in the UK and for mothers of Black and American Indian children in the US.

Table 3 shows the relationship between children's mean BMI, race/ethnicity and a range of explanatory factors in the UK and US. In the base model in the UK, Indian and Pakistani children had significantly lower BMI (coefficient: −0.69, s.e.=0.12; coefficient: −0.40, s.e.=0.09) and Black Caribbean and Black African children had higher BMI (coefficient: 0.54, s.e.=0.17; coefficient: 0.50, s.e.=0.15) than White children. For the US, Asian Indian children had lower BMI (coefficient: −0.58, s.e.=0.26), and Mexican, other Hispanic and American Indian children had significantly higher BMI (coefficient: 0.41, s.e.=0.14; coefficient: 0.39, s.e.=0.19; coefficient: 0.87, s.e.=0.29) than White children. Adjustment for sociodemographic characteristics amplified the relationship among Pakistani children, and slightly attenuated the relationship for Black Caribbean and Black African children (panel A; model 1). Adjustment for sociodemographic factors reduced the disparities for Mexican, other Hispanic and Asian Indian children to nonsignificance and attenuated estimates for American Indian children (panel B; model 1). Adjustment for markers of cultural tradition and family routines amplified the association for Indian and Pakistani children and attenuated the association for Black Caribbean and Black African children (panel A; models 2 and 3). In the US, adjustment for these markers made no difference to estimates for American Indian children (models 2 and 3). In both countries, adjustment for a marker of nutrition had no effect (model 4). Adjustment for maternal BMI attenuated estimates for Indian, Pakistani and Black Caribbean children, and Black African children no longer differed from White children (panel A; model 5). Adjustment for maternal BMI did not explain the disadvantage among American Indian children (panel B; model 5). Fully adjusted models showed significant advantages for Indian and Pakistani children (coefficient: −0.72, s.e.=0.13; coefficient: −0.56, s.e.=0.13), a significant disadvantage for Black Caribbean children (coefficient: 0.38, s.e.=0.17) and the disadvantage among Black African children became statistically non-significant (coefficient: 0.17, s.e.=0.14) (panel A; model 6). The disadvantage among American Indian children remained after adjusting for all explanatory factors (coefficient: 0.69, s.e.=0.28; panel B; model 6), but the disadvantage for Mexican and other Hispanic children and the advantage for Asian Indian children disappeared.

Table 4 illustrates the odds of obesity and overweight across racial/ethnic groups in the UK. The first panel presents results for the odds of obesity. In the base model, Bangladeshi, Black Caribbean and Black African children, as compared to White children, were significantly more likely to be obese (odds ratio, OR=2.0, confidence interval, CI=1.2–3.2; OR=2.3, CI=1.7–3.3; and OR=2.4, CI=1.6–3.7, respectively). Adjustment for sociodemographic measures attenuated the difference between Bangladeshi and White children to non-significant levels, but did not completely explain the differences for Black Caribbean and Black African children (model 1). Adjustment for markers of cultural tradition, family routines, a marker of nutrition and maternal BMI did not fully explain the Black Caribbean and Black African disadvantage (models 2–5), although adjustment for cultural factors did most to attenuate the odds for these two groups. In fully adjusted models, the Black Caribbean disadvantage remained unexplained whereas the Black African disadvantage was no longer apparent (OR=1.7, CI=1.1–2.6; OR=1.3, CI=0.8–2.0, respectively). The only group to have significantly reduced odds of obesity was Pakistani children who were 40% less likely to be obese than White children.

The second panel of Table 4 shows the odds of overweight. In the unadjusted model, as compared to White children, Indian children were significantly less likely (OR=0.7, CI=0.5–1.0) and Black African children were more likely to be overweight (OR=1.8, CI=1.3–2.3). Adjustment for sociodemographic, cultural and family routine factors, and a marker of nutrition had no effect (models 1–4). Adjusting for maternal BMI explained the Indian advantage and partially explained the Black African disadvantage (model 5). In fully adjusted models (model 6), the Black African disadvantage remained unexplained (OR=1.4, CI=1.0–1.9).

The corresponding results for US children are shown in Table 5. In base models, Mexican, other Hispanic and American Indian children were 50–70% more likely to be obese than White children (OR=1.6, CI=1.2–2.0; OR=1.5, CI=1.0–2.1; and OR=1.7, CI=1.0–3.0, respectively). These disadvantages were fully explained after adjustment for sociodemographic factors (model 1). In the second panel, Mexican children were more likely to be overweight than their White peers (OR=1.2, CI=1.0–1.5) in base models. This disadvantage was explained on adjustment for sociodemographic factors (model 1).

## Discussion

We used two national cohort studies to investigate race/ethnic disparities in children's mean BMI and odds of obesity and overweight at 5 years of age and examine factors that influence these patterns in the UK and US. In the UK, we found Black Caribbean children were more likely to be obese and Black African children to be overweight than White children. These differences were not explained by potential explanatory factors as operationalized here. Pakistani children had lower odds of obesity. In the US, Mexican, other Hispanic and American Indian children were more likely to be obese than White children, but these differences disappeared after adjusting for sociodemographic markers.

High BMI, obesity and overweight, as markers of health disadvantage, are associated with minority status in both countries, but the factors that explain the race/ethnic disparities appear to differ between the UK and US. In the UK, sociodemographic measures completely explained the health disadvantages for Bangladeshi children but only partially explained Black Caribbean and Black African disparities. Cultural markers explained 11–30% of the higher odds of obesity among Black Caribbean and Black African children, respectively. The evidence regarding mothers' migration status and its association with the odds of obesity is mixed, and there is a paucity of research in the UK context,^21^ suggesting future research on maternal migration and children's health may be of merit. In the US, however, adjustment for sociodemographic factors explained the disparities for Mexican, other Hispanic and American Indian children. Empirical evidence supports high socioeconomic status, as measured by income and education, to be protective against obesity.^1,5^ However, there is some evidence that a negative socioeconomic gradient is moderated by race/ethnicity: particularly, this gradient may not exist for Black and Hispanic children in the US^1^ or may vary by the extent of economic development of a mother's country of origin.^18^

The influence of family routines, children's nutrition and maternal BMI had little to no influence in explaining racial/ethnic differences in the risk of overweight and obesity in the UK and US. There is some evidence that bedtimes are linked to the risk of overweight and obesity in the UK models. This is supported by several studies demonstrating the link between shorter durations of sleep and bedtimes to the greater risk of high BMI and overweight status, both in the short-term and long-term.^47, 48, 49^ Although we found no evidence of the influence of children's nutrition, our study was limited to one marker of nutrition due to data constraints. Research has documented multiple markers of children's diet, including fast food consumption, unhealthy snacks and regularity of breakfast, to be predictive of children's risk of overweight and obesity.^19^ Thus, we cannot conclude children's nutrition is not relevant to racial and ethnic inequalities in children's early childhood health based on our findings. However, it would be fruitful for future research to examine more detailed markers of children's nutrition to understand the development of racial/ethnic disparities in children's risk of overweight and obesity. We found some associations between maternal BMI and children's risk of overweight and obesity in the UK which is substantiated by previous literature.^4,5^

Our findings confirm prior reports of race/ethnic disparities. There is limited research examining racial/ethnic patterning in child overweight and obesity in the UK context, particularly during early childhood, and previous studies do not consistently disaggregate ‘South Asian' and ‘Black' groups.^4,7^ Findings from other studies that aggregate children from a South Asian background suggest a higher risk of obesity and overweight.^4,7,50^ However, using disaggregated South Asian classifications reveals variations in the odds of obesity and overweight; we find Pakistani children to have lower odds of obesity. Another UK study reported lower risk of overweight and obesity among Pakistani children.^5^ Although we find a lower risk of obesity for Pakistani children, this group faces a greater risk of insulin resistance, a predictor of cardiovascular disease.^51^ In addition, recent comparative work reveals that Asian children (aggregate group) may be at risk for obesity at school-age years despite having a healthy weight in early childhood.^31^ We find Black Caribbean children to have higher odds of obesity and Black African children to have higher odds of overweight, while other studies suggest a disadvantage for an aggregated Black group.^4,5,52^ The disadvantage among Mexican, other Hispanic and American Indian children in the US is consistent with findings from other studies.^1,4,24,53^ Specifically, we find nearly 50% greater odds of obesity for Mexican and other Hispanic children, while another study, using an aggregate Hispanic group, reported a two-fold risk of overweight or obesity.^24^ Thus aggregations of racial/ethnic groups may obscure important differences in socioeconomic profiles and migratory histories and potentially misattribute health advantages/disadvantages.

Although our paper used a wide range of explanatory factors, large unexplained disadvantages remain in the UK models for Black Caribbean and Black African children. Black children in the US models do not show similar disadvantages, findings that are surprising given the similar contemporary socioeconomic profiles and shared migration histories between Black Caribbean individuals in the UK and Black Americans. Despite these similarities, income inequality is lower in the UK than in the US.^28^ Particularly, the magnitude of socioeconomic differentials between Black Caribbean individuals and their White counterparts in the UK is less pronounced than that of Black American individuals and White individuals in the US. Thus, in our UK models, sociodemographic measures do not reduce disparities between Black African and Black Caribbean and White children to nonsignificance while adjustment for such factors reduce racial/ethnic disparities in our US models to nonsignificance. The international comparison in this paper also revealed the health advantages for Pakistani children in the UK (and Indian children in unadjusted models) were not apparent for Asian Indians in the US. These results are revealing because nearly two-thirds of Pakistani people in the UK are living in households in the lowest income tertile.^54^ The finding that not all advantages and disadvantages are shared by similar race/ethnic groups in the UK and US merits additional investigation and is of public health significance.

Our results are consistent with previous research comparing racial and ethnic disparities in early childhood health in the UK and US.^4,31,39^ Similar to earlier studies, our analyses demonstrate health disadvantages for ethnic minorities, with socioeconomic factors and migration status influencing the risk of child overweight. However, previous comparative research used aggregate ethnic classifications, making it difficult to compare such studies to describe which racial and ethnic minorities experience health disadvantages or advantages. Despite the differences in ethnic classifications, it is revealing that existing studies and our analyses show that racial and ethnic disparities are of similar magnitude between the two countries, and that these disparities remain after controlling for a host of explanatory factors. This is surprising given the more generous social welfare system and the universal access to health care in the UK in contrast to the uneven access to health care in the US.^28,55^ It is possible that quality of healthcare is associated with minority status suggesting that policy interventions need to ensure quality of health care to reduce racial and ethnic disparities.

Our findings suggest obesity prevention should begin in early in childhood. Prevention programs should work with parents and children to encourage healthful eating and physical behaviors.^56^ School-based interventions, for example, provision of nutritious food, opportunities for physical activity and obesity-related health services may be beneficial for at-risk children.^57^ Our work, along with previous findings,^5^ suggest that high parental BMI adversely influences the risk of childhood overweight and obesity. Thus helping parents maintain a healthy weight may have a positive impact on child outcomes as well as addressing adult obesity, a policy goal for both the US and UK.^58,59^ Emerging evidence also suggests supporting postnatal practices such as breastfeeding to reduce disparities for the risk of overweight and obesity.^60^

This study is not without limitations. First, we used cross-sectional analyses and this limits our ability to make causal inferences. A recent study has demonstrated longitudinal associations between race/ethnicity and high BMI.^10^ Second, BMI is an imperfect proxy for adiposity as it does not differentiate between lean and fat mass and it has been suggested that BMI may not be reliable for studies of ethnic differences in body composition.^61^ However, we were unable to use other types of anthropometric measurements (for example, waist circumference).^14^ However, BMI is a widely accepted measure of body fatness and a widely used indicator of obesity in populations.^37^ Another limitation is that we were confined to a single marker of children's nutrition in efforts to harmonize our UK and US datasets. A broader range of nutrition markers may have provided greater explanatory power in our statistical models. Further, our explanatory model does not address the influence of adverse environments, including discrimination, poor housing and pollution, which have been linked to health inequalities.^62^ Lastly, we were unable to consider community level covariates, such as neighborhood context or physical and built environmental factors (for example, access to recreational facilities, healthy food options and neighborhood safety), which have been identified as influential on disparities in childhood obesity and overweight.^15,63,64^

## Conclusion

Our work adds to the body of literature investigating racial/ethnic patterning of BMI, overweight and obesity in an internationally comparative context during early childhood. In the UK, compared to White children, we have shown Black Caribbean children to have higher odds and Pakistani children to have lower odds of obesity, and increased odds of overweight for Black African children. In the US, Mexican, other Hispanic and American Indian children have increased odds of obesity. Socioeconomic disadvantage did most to explain disparities in the US, while a combination of socioeconomic, cultural and family factors did most to explain differences in the UK context. In order to reduce racial/ethnic health disparities in early childhood, future public health interventions in the UK and US need to consider the different socioeconomic and cultural profiles of race/ethnic groups.

## Figures and Tables

**Table 1:** Children's BMI and obesity and the distribution of explanatory factors by race/ethnicity: UK

	*White (*n*=15 003)*	*Indian (*n*=518)*	*Pakistani (*n*=926)*	*Bangladeshi (*n*=376)*	*Black Caribbean (*n*=487)*	*Black African (*n*=459)*	*Other (*n*=511)*
*Child characteristics*							
BMI, mean (s.e.)	16.4 (0.02)	15.7 (0.12)	16.0 (0.09)	16.3 (0.19)	16.9 (0.17)	16.9 (0.15)	16.1 (0.11)
Obesity, %	5.5	4.7	6.5	10.7	11.4	11.1	6.2
Age in months (s.e.)	62.5 (0.05)	62.7 (0.18)	62.5 (0.13)	62.7 (0.22)	62.5 (0.18)	62.6 (0.17)	62.6 (0.20)
Child is male	51.5	52.8	49.6	48.5	53.6	50.6	48.8
							
*Sociodemographic factors*							
Equivalized household income							
Lowest quintile	16.4	15.5	48.1	57.4	37.2	40.4	25.7
Second quintile	18.8	24.4	33.3	26.8	23.2	20.2	21.2
Third quintile	21.0	18.8	11.2	8.5	17.5	14.5	14.9
Fourth quintile	21.6	21.1	3.5	4.7	11.1	11.5	21.5
Highest quintile	22.2	20.2	3.9	2.5	11.0	13.4	16.7
Education							
Less than O level	10.4	10.4	27.2	32.6	15.4	19.1	17.0
O level	24.2	13.0	21.1	23.2	26.0	11.5	9.6
A level	16.1	11.3	12.9	12.0	14.0	9.3	12.1
Degree or higher	48.2	61.1	28.0	20.1	42.4	51.1	56.6
Other qualifications	1.1	4.1	10.8	12.1	2.3	8.9	4.6
Single parent	18.3	8.7	13.9	5.8	47.8	41.9	21.8
Mother's employment							
Full-time	18.3	25.6	3.9	2.2	24.7	29.5	25.7
Part-time	42.6	35.1	12.3	12.7	26.7	14.6	25.1
Not working	38.3	38.6	82.9	84.5	45.9	54.7	49.1
No mother in household	0.8	0.7	0.8	0.6	2.4	1.3	0.4
							
*Cultural factors*							
Language spoken at home is primarily English	98.8	62.5	41.4	18.7	98.8	69.5	70.5
Migrant generation							
First generation	4.6	49.4	58.5	89.7	13.0	65.6	58.2
Second generation	1.6	42.0	36.6	7.5	34.4	16.7	8.9
Third generation	93.8	8.6	4.9	2.7	52.7	17.8	32.9
							
*Family routines*							
Always/usually has regular bedtimes	91.3	86.3	85.0	88.1	81.0	79.5	86.5
Bedtime on weekdays							
None given	4.3	4.2	7.2	3.6	8.2	9.5	6.4
Before 1930 h	26.8	11.3	4.5	2.9	14.3	10.9	13.3
1930–1959 h	33.9	16.9	8.5	5.0	26.9	14.3	21.1
2000–2029 h	23.7	30.2	28.6	19.7	29.5	27.0	26.0
2030–2059 h	7.4	18.8	19.4	22.4	10.6	21.5	16.7
2100 h or later	3.9	18.6	31.7	46.4	10.5	16.8	16.4
							
*Nutrition*							
How many portions of fruit per day							
None	3.9	4.4	5.5	11.0	5.0	3.8	3.1
One	14.3	25.2	28.8	35.1	19.5	19.5	21.6
Two	26.6	30.3	34.7	31.3	31.6	33.3	27.7
Three or more	55.2	40.1	31.0	22.6	43.9	43.3	47.6
Mother's BMI, mean (s.e.)	25.3 (0.07)	24.3 (0.23)	25.7 (0.23)	25.4 (0.32)	25.9 (0.29)	27.7 (0.38)	24.6 (0.26)

Abbreviations: A level, advanced level; BMI, body mass index; MCS, Millennium Cohort Study; O level, ordinary level. Figures are percentages that are weighted with overall weights from MCS 1. Sample sizes are unweighted.

**Table 2:** Children's BMI and obesity, and the distribution of explanatory factors by race/ethnicity: US

	*White (*n*=3300)*	*Black (*n*=1400)*	*Mexican (*n*=1250)*	*Other Hispanic (*n*=650)*	*American Indian (*n*=300)*	*Asian Indian (*n*=250)*	*East Asian (*n*=550)*	*Southeast Asian (*n*=350)*	*Other (*n*=800)*
*Child characteristics*									
BMI, mean (s.e.)	16.40 (0.08)	16.66 (0.12)	16.81 (0.12)	16.78 (0.17)	17.26 (0.29)	15.81 (0.26)	16.30 (0.19)	16.42 (0.21)	16.76 (0.23)
Obesity, %	11.3	13.5	15.9	15.3	16.6	7.4	9.5	12.1	14.8
Age in months (s.e.)	64.87 (0.08)	64.88 (0.08)	64.88 (0.14)	64.91 (0.20)	64.92 (0.41)	64.43 (0.37)	64.95 (0.22)	65.14 (0.25)	65.01 (0.25)
Child is male	51.8	50.6	51.3	49.4	53.3	51.0	50.2	57.4	47.8
									
*Sociodemographic factors*									
Equivalized household income									
Lowest quintile	13.8	34.0	25.6	21.0	34.0	9.0	9.8	18.3	19.8
Second quintile	16.4	20.6	29.8	20.4	27.2	12.0	13.4	19.6	17.9
Third quintile	19.7	19.6	21.6	24.0	15.8	20.0	14.3	24.1	19.7
Fourth quintile	23.8	14.5	13.7	17.8	16.5	21.7	21.7	22.6	22.4
Highest quintile	26.2	11.3	9.2	16.8	6.6	37.3	40.8	15.3	20.2
Education									
Less than high school	2.3	11.7	20.6	10.6	7.2	0.00	2.6	12.4	4.1
High school/GED	13.7	31.9	33.6	24.4	24.4	7.0	7.1	15.0	16.8
Some college	35.8	38.4	33.0	36.5	56.3	10.0	9.9	35.8	44.7
Bachelor's degree or higher	48.2	18.0	12.8	29.2	12.1	82.5	80.4	36.8	34.4
Single parent	14.1	56.4	21.9	29.4	34.0	6.5	4.4	19.4	32.9
Mother's employment									
Full-time	40.3	50.8	38.7	49.8	46.4	33.9	48.0	48.7	45.1
Part-time	22.2	16.1	16.3	16.3	13.5	12.4	15.8	17.8	16.8
Not working	36.5	33.0	44.6	33.0	38.1	53.6	35.9	32.0	36.5
No mother in household	1.0	0.00	0.5	0.9	2.0	0.00	0.00	1.3	1.7
									
*Cultural factors*									
Language spoken at home is primarily English	100.0	99.5	56.9	72.7	100.0	94.0	87.6	85.1	100.0
Migrant generation									
First generation	1.7	4.5	22.3	23.6	1.5	22.2	21.5	31.7	8.6
Second generation	3.0	6.9	36.6	27.6	0.00	73.7	68.9	46.1	4.8
Third generation	95.3	88.6	41.1	48.7	98.5	4.1	9.6	22.2	86.6
									
*Family routines*									
Always/usually has regular bedtimes	96.1	95.3	90.9	93.0	94.9	96.5	93.3	94.5	94.0
Bedtime on weekdays									
Before 1930 h	2.7	1.4	1.8	2.2	5.0	0.00	0.9	2.4	2.2
1930–1959 h	9.1	3.6	3.3	5.1	4.7	5.0	2.9	3.5	8.4
2000–2029 h	28.7	21.5	21.5	23.1	19.8	21.9	15.3	18.0	23.5
2030–2059 h	27.2	26.4	24.3	27.2	22.3	21.2	17.3	18.7	26.0
2100 h or later	32.2	47.1	49.1	42.4	48.2	52.1	63.6	57.4	39.9
									
*Nutrition*									
How many portions of fruit per day									
None	16.6	18.5	14.8	17.1	18.8	15.9	14.5	14.6	17.0
One	43.7	42.9	38.9	42.0	40.3	57.2	48.2	50.1	46.0
Two	26.6	21.2	26.4	25.1	24.2	18.4	27.8	21.0	22.5
Three or more	13.1	17.4	19.9	15.9	16.6	8.5	9.5	14.3	14.5
Mother's BMI, mean (s.e.)	27.38 (0.13)	30.10 (0.20)	28.72 (0.24)	28.18 (0.33)	29.71 (0.82)	25.69 (0.31)	23.44 (0.42)	25.44 (0.42)	28.57 (0.45)

Abbreviations: BMI, body mass index; GED, General Education Diploma; IES, Institute of Education Sciences; NCES, National Center for Education Statistics. Percentages are weighted with respondent weights from the 9-month survey. Sample sizes are unweighted and rounded to the nearest 50. Percentages for cells containing fewer than four observations have been set to 0 per NCES/IES security guidelines and have been included in the closest cell/category.

**Table 3:** Multivariate linear regressions predicting BMI in the UK and US

	*Base model: ethnicity, age and gender*^a^	*Model 1: sociodemographic*^b^	*Model 2: sociodemographic+cultural factors*^c^	*Model 3: sociodemographic+family routines*^d^	*Model 4: sociodemographic+nutrition*^e^	*Model 5: sociodemographic+mother's BMI*	*Model 6: fully adjusted model*
*Panel A: UK*							
Indian	−0.69 (0.12)***	−0.70 (0.12)***	−0.76 (0.13)***	−0.75 (0.13)***	−0.69 (0.12)***	−0.61 (0.12)***	−0.72 (0.13)***
Pakistani	−0.40 (0.09)***	−0.45 (0.10)***	−0.52 (0.13)***	−0.51 (0.10)***	−0.44 (0.10)***	−0.43 (0.10)***	−0.56 (0.13)***
Bangladeshi	−0.07 (0.19)	−0.13 (0.19)	−0.18 (0.21)	−0.19 (0.20)	−0.12 (0.19)	−0.08 (0.19)	−0.20 (0.21)
Black Caribbean	0.54 (0.17)**	0.48 (0.17)**	0.45 (0.18)*	0.45 (0.17)**	0.49 (0.17)**	0.44 (0.17)**	0.38 (0.17)*
Black African	0.50 (0.15)**	0.43 (0.15)**	0.40 (0.16)*	0.37 (0.15)*	0.44 (0.15)**	0.27 (0.14)	0.17 (0.14)
Other	−0.22 (0.11)*	−0.25 (0.11)*	−0.28 (0.12)*	−0.29 (0.11)**	−0.25 (0.11)*	−0.19 (0.11)	−0.26 (0.12)*
*N*	18 280	18 280	18 280	18 280	18 280	18 280	18 280
							
*Panel B: US*							
Black	0.26 (0.15)	0.17 (0.16)	0.18 (0.17)	0.16 (0.17)	0.17 (0.16)	0.05 (0.17)	0.05 (0.17)
Mexican	0.41 (0.14)**	0.30 (0.16)	0.20 (0.18)	0.28 (0.16)	0.31 (0.16)	0.27 (0.16)	0.12 (0.18)
Other Hispanic	0.39 (0.19)*	0.32 (0.19)	0.26 (0.20)	0.31 (0.19)	0.32 (0.19)	0.30 (0.19)	0.19 (0.20)
American Indian	0.87 (0.29)**	0.76 (0.30)*	0.77 (0.30)*	0.76 (0.30)*	0.76 (0.30)*	0.68 (0.28)*	0.69 (0.28)*
Asian Indian	−0.58 (0.26)*	−0.50 (0.27)	−0.45 (0.31)	−0.51 (0.26)	−0.50 (0.27)	−0.44 (0.26)	−0.49 (0.31)
Southeast Asian	0.02 (0.22)	−0.04 (0.23)	−0.04 (0.26)	−0.05 (0.23)	−0.04 (0.23)	0.09 (0.23)	0.02 (0.26)
East Asian	−0.09 (0.21)	−0.05 (0.21)	−0.03 (0.27)	−0.07 (0.22)	−0.05 (0.21)	0.14 (0.21)	0.07 (0.28)
Other	0.36 (0.24)	0.33 (0.24)	0.33 (0.24)	0.33 (0.24)	0.33 (0.24)	0.28 (0.24)	0.27 (0.24)
*N*	8850	8850	8850	8850	8850	8850	8850

Abbreviation: BMI, body mass index.

***P<0.001, **P<0.01 and *P<0.05.

All estimates are weighted and adjusted for design effects.

a White is the reference group.

b Adusted for child age, child gender, income, education, single parenthood and mother's employment.

c Adjusted for language spoken at home and migrant generation.

d Adusted for regular bedtime on weekdays and what bedtime on weekdays.

e Adusted for how many portions of fruit per day.

**Table 4:** Odds ratios (95% CI) of obesity and overweight by race/ethnicity^a^: UK

	*Base model: ethnicity, age and gender*^b^	*Model 1: sociodemographic*^c^	*Model 2: sociodemographic+language and migrant generation*^d^	*Model 3: sociodemographic+family routines*^e^	*Model 4: sociodemographic+nutrition*^f^	*Model 5: sociodemographic+mother's BMI*	*Model 6: fully adjusted model*
*Obesity*							
Indian	0.79 (0.43–1.47)	0.77 (0.41–1.41)	0.57 (0.30–1.10)	0.69 (0.37–1.30)	0.76 (0.41–1.41)	0.88 (0.47–1.64)	0.60 (0.30–1.17)
Pakistani	1.13 (0.82–1.56)	0.93 (0.65–1.32)	0.66 (0.42–1.04)	0.81 (0.56–1.18)	0.92 (0.65–1.31)	0.95 (0.67–1.36)	0.60* (0.37–0.96)
Bangladeshi	1.98** (1.24–3.15)	1.56 (0.95–2.54)	1.09 (0.60–1.98)	1.37 (0.82–2.27)	1.55 (0.95–2.52)	1.70* (1.04–2.78)	1.04 (0.57–1.90)
Black Caribbean	2.34*** (1.65–3.32)	2.07*** (1.43–2.99)	1.88** (1.25–2.81)	1.95*** (1.35–2.83)	2.06*** (1.43–2.98)	2.00*** (1.38–2.91)	1.72* (1.14–2.61)
Black African	2.43*** (1.59–3.70)	2.10*** (1.36–3.24)	1.70* (1.08–2.70)	1.89** (1.20–2.96)	2.10*** (1.36–3.24)	1.72* (1.12–2.64)	1.25 (0.79–1.99)
Other	1.08 (0.67–1.74)	1.01 (0.62–1.63)	0.84 (0.50–1.43)	0.93 (0.57–1.51)	1.01 (0.63–1.63)	1.11 (0.68–1.80)	0.84 (0.49–1.45)
							
*Overweight*							
Indian	0.71* (0.52–0.97)	0.71* (0.52–0.96)	0.69* (0.48–0.98)	0.66* (0.48–0.92)	0.72* (0.52–0.98)	0.76 (0.56–1.04)	0.70 (0.48–1.01)
Pakistani	0.78 (0.59–1.02)	0.77 (0.58–1.02)	0.77 (0.55–1.07)	0.71* (0.53–0.96)	0.78 (0.59–1.03)	0.78 (0.59–1.04)	0.72 (0.51–1.01)
Bangladeshi	0.82 (0.56–1.22)	0.81 (0.55–1.20)	0.87 (0.56–1.34)	0.75 (0.50–1.11)	0.83 (0.56–1.23)	0.85 (0.57–1.26)	0.84 (0.54–1.30)
Black Caribbean	1.37 (0.98–1.91)	1.33 (0.95–1.85)	1.26 (0.89–1.78)	1.28 (0.92–1.79)	1.33 (0.96–1.86)	1.29 (0.93–1.80)	1.19 (0.83–1.69)
Black African	1.76*** (1.33–2.33)	1.69*** (1.27–2.26)	1.71*** (1.26–2.31)	1.58** (1.19–2.11)	1.70*** (1.28–2.27)	1.49** (1.13–1.98)	1.40* (1.04–1.88)
Other	0.84 (0.60–1.18)	0.82 (0.58–1.16)	0.83 (0.57–1.22)	0.78 (0.55–1.11)	0.82 (0.58–1.16)	0.86 (0.60–1.23)	0.83 (0.56–1.23)
*N*	18 280	18 280	18 280	18 280	18 280	18 280	18 280

Abbreviations: BMI, body mass index; CI, confidence interval.

***P<0.001, **P<0.01 and *P<0.05.

All estimates are weighted with analytic weights.

a Children who are normal weight are in the reference group.

b White is the reference group.

c Adusted for child age, child gender, income, education, single parenthood and mother's employment.

d Adjusted for language spoken at home and migrant generation.

e Adjusted for what bedtime on weekdays. Family routines do not adjust for regular bedtimes on weekdays.

f Adjusted for how many portions of fruit per day.

**Table 5:** Odds ratios (95% CI) of obesity and overweight by race/ethnicity^a^: US

	*Base model: ethnicity, age and gender*^b^	*Model 1: sociodemographic*^c^	*Model 2: sociodemographic+language and migrant generation*^d^	*Model 3: sociodemographic+family routines*^e^	*Model 4: sociodemographic+nutrition*^f^	*Model 5: sociodemographic+mother's BMI*	*Model 6: fully adjusted model*
*Obesity*							
Black	1.27 (0.92–1.73)	1.05 (0.75–1.49)	1.05 (0.74–1.49)	1.04 (0.73–1.48)	1.05 (0.75–1.48)	0.95 (0.67–1.35)	0.93 (0.65–1.34)
Mexican	1.55** (1.20–2.01)	1.26 (0.94–1.69)	1.15 (0.82–1.63)	1.23 (0.91–1.66)	1.26 (0.94–1.69)	1.24 (0.92–1.67)	1.08 (0.76–1.53)
Other Hispanic	1.47* (1.02–2.13)	1.30 (0.89–1.91)	1.22 (0.81–1.84)	1.28 (0.87–1.89)	1.30 (0.89–1.91)	1.29 (0.88–1.90)	1.15 (0.76–1.75)
American Indian	1.73* (1.00–2.99)	1.46 (0.84–2.54)	1.47 (0.84–2.55)	1.46 (0.84–2.54)	1.46 (0.84–2.54)	1.37 (0.81–2.32)	1.37 (0.81–2.32)
Asian Indian	0.59 (0.22–1.58)	0.67 (0.25–1.81)	0.66 (0.24–1.80)	0.67 (0.25–1.79)	0.67 (0.25–1.79)	0.72 (0.27–1.92)	0.64 (0.24–1.75)
Southeast Asian	1.11 (0.68–1.81)	1.00 (0.60–1.67)	0.95 (0.53–1.72)	0.99 (0.59–1.66)	1.00 (0.60–1.67)	1.13 (0.67–1.90)	1.01 (0.56–1.81)
East Asian	0.81 (0.45–1.46)	0.88 (0.48–1.61)	0.85 (0.41–1.76)	0.86 (0.46–1.59)	0.88 (0.48–1.62)	1.05 (0.57–1.93)	0.93 (0.45–1.93)
Other	1.43 (0.88–2.31)	1.35 (0.83–2.20)	1.34 (0.82–2.19)	1.34 (0.82–2.19)	1.35 (0.83–2.20)	1.30 (0.79–2.13)	1.27 (0.77–2.08)
							
*Overweight*							
Black	1.17 (0.93–1.47)	1.12 (0.87–1.44)	1.12 (0.87–1.44)	1.12 (0.87–1.45)	1.13 (0.88–1.45)	1.05 (0.82–1.36)	1.04 (0.81–1.35)
Mexican	1.22* (0.99–1.49)	1.18 (0.95–1.48)	1.09 (0.84–1.43)	1.17 (0.94–1.47)	1.19 (0.95–1.48)	1.17 (0.93–1.46)	1.05 (0.80–1.38)
Other Hispanic	1.21 (0.85–1.73)	1.19 (0.82–1.72)	1.12 (0.77–1.64)	1.18 (0.81–1.71)	1.19 (0.82–1.73)	1.18 (0.81–1.71)	1.08 (0.74–1.59)
American Indian	1.44 (0.93–2.22)	1.39 (0.89–2.17)	1.39 (0.89–2.17)	1.38 (0.88–2.15)	1.39 (0.89–2.17)	1.33 (0.85–2.08)	1.33 (0.85–2.08)
Asian Indian	0.81 (0.49–1.35)	0.83 (0.49–1.39)	0.79 (0.45–1.39)	0.83 (0.49–1.39)	0.83 (0.49–1.39)	0.85 (0.51–1.43)	0.78 (0.44–1.37)
Southeast Asian	1.14 (0.76–1.71)	1.15 (0.76–1.72)	1.08 (0.68–1.72)	1.14 (0.76–1.72)	1.15 (0.77–1.73)	1.23 (0.82–1.84)	1.12 (0.71–1.78)
East Asian	0.99 (0.64–1.52)	1.00 (0.64–1.55)	0.94 (0.56–1.59)	0.99 (0.64–1.54)	1.00 (0.64–1.55)	1.10 (0.71–1.71)	0.99 (0.60–1.66)
Other	1.26 (0.86–1.83)	1.24 (0.85–1.82)	1.24 (0.84–1.81)	1.24 (0.84–1.82)	1.24 (0.85–1.83)	1.21 (0.83–1.78)	1.19 (0.81–1.75)
*N*	8850	8850	8850	8850	8850	8850	8850

Abbreviations: BMI, body mass index; CI, confidence interval.

**P<0.01 and *P<0.05.

All estimates are weighted with analytic weights.

a Children who are normal weight are in the reference group.

b White is the reference group.

c Adusted for child age, child gender, income, education, single parenthood and mother's employment.

d Adjusted for language spoken at home and migrant generation.

e Adjusted for what bedtime on weekdays. Family routines do not adjust for regular bedtimes on weekdays.

f Adjusted for how many portions of fruit per day.
